# A new technique to determine vertical dimension of occlusion from anthropometric measurement of interpupillary distance

**DOI:** 10.4317/jced.51671

**Published:** 2014-10-01

**Authors:** Ruchi Ladda, Vikrant O. Kasat, Aruna J. Bhandari

**Affiliations:** 1 MDS, Assistant Professor. Department of Prosthodontics, Rural Dental College, Loni; 2 MDS, Associate Professor. Department of Oral Medicine and Radiology, Rural Dental College, Loni; 3 MDS, Professor. Department of Prosthodontics, Rural Dental College, Loni

## Abstract

Background: A number of techniques are being practiced for the evaluation of VDO, but none of them is scientifically more accurate than other. Each method advocated has its own limitations. Objectives: The purpose of this study was to find correlation between vertical dimension of occlusion (VDO) and interpupillary distance (IPD). Material and Methods: A cross-sectional study was conducted on 400 dentate subjects comprising of 200 males and 200 females. Anthropometric measurement of VDO was recorded clinically using modified digital vernier caliper. Also, a standardized digital photograph of face was generated from the frontal aspect using a digital camera for the measurement of IPD in millimeters. Correlation between VDO and IPD was studied using Spearman’s coefficient. For the execution of regression command and preparation of prediction equations to estimate VDO, Statistical Package for Social Sciences (SPSS) Software Version 11.5 was used. Results: VDO and IPD was more in males compared to females. VDO was significantly and positively correlated with IPD only in males whereas females showed a weak correlation. Hence, regression equation was derived only for males. VDO estimation using regression equation for IPD had a standard error of ± 3.94 in males. Conclusions: Since the variations between VDO and IPD are within the range of 2-4 mm, VDO prediction through this method is reliable and reproducible for male patients. Also, the method is simple, economic, and non invasive; hence it could be recommended for everyday practice to determine vertical dimension of occlusion in case of male patients.

** Key words:**Anthropometry, interpupillary distance, jaw relation, vertical dimension of occlusion.

## Introduction

Glossary of Prosthodontic Terms defines vertical dimension as the distance between the two selected anatomic or marked points [usually one on the tip of the nose and the other upon the chin], one on a fixed and one on a movable member (1).

Importance of establishing an appropriate lower facial height [when lost] cannot be overlooked because if VDO is registered too high or too low, it would deteriorate the existing patient’s condition. Although many techniques exist for the evaluation of VDO, none of them is scientifically more accurate than other and each method has its own limitations (2). They are either tedious, time consuming, or expose patients to radiation (3). They may require equipments like lateral cephalographic unit (4) or electromyographic machine (5) that is not available in most of the dental clinics.

In the past, VDO has been correlated with various anthropometric measurements like the distance from the outer canthus of one eye to the inner canthus of the other eye, vertical height of the ear, twice the length of one eye, and vertical length of nose at the midline. Similarly we have earlier correlated original VDO to length of fingers (2).

In line with these observations, we designed this study to find correlation between VDO and IPD so as to explore the possibility of another method for determination of VDO. The research hypothesis was that there would be a significant relationship between the VDO and IPD.

## Material and Methods

For this study, 400 physically healthy dentate subjects comprising of 200 males and 200 females with the age range of 20 to 30 years having no deformity of eyes were selected randomly from Rural Dental College and Hospital, Loni. All the participants had eugnathic jaw relationship and a definite centric stop with at least 28 fully erupted, periodontally sound teeth in both jaws. Subjects with the following conditions were excluded from the study: open bite or deep bite cases, teeth anomalies, attrition, extensive prosthesis or restorations in the oral cavity, temporomandibular joint disorders or any other pathology in the maxillofacial region, history of trauma, orthodontic treatment or orthognathic surgery.

Clearance from the Institutional Ethical committee was obtained. All subjects provided written informed consent to participate in the study. Anthropometric measurement of VDO was recorded clinically in millimeters using a modified digital vernier caliper with an accuracy of 0.01mm as described by us in an earlier study (2).

Also, a standardized digital photograph of face was generated from the frontal aspect using a digital camera for the measurement of IPD in millimeters.

**Figure 1:**
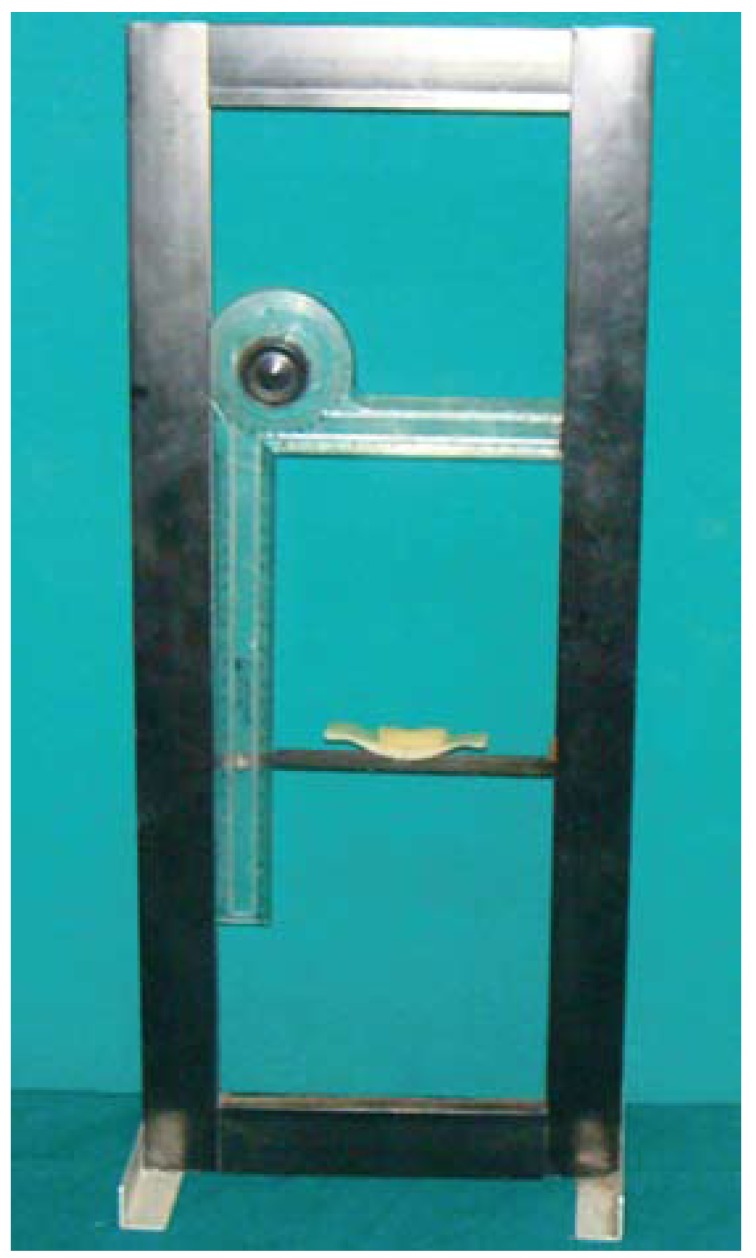
Modified set square with movable working scales.

**Figure 2:**
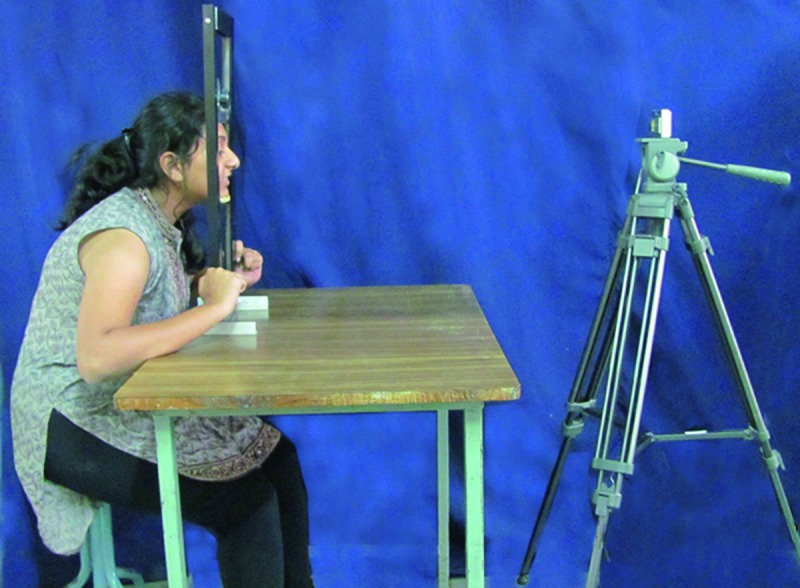
Standardised photographic set-up.

- Photographic Methodology:

To shoot photographs for the measurement of IPD a modified set square with movable working scales [Omega mini drafter, Art no 1954, Altop Industries, Mumbai, India] was used (Fig. 1). The working scales provided a measurable relationship between the actual and image dimensions. A digital camera was set up at a fixed distance on a tripod which was adjusted to the patient’s height. To standardize the head position, subjects were instructed to position their face in a modified set square instrument with chin rest guiding the chin position. They were then asked to look straight and photograph was clicked (Fig. 2). All the photographs were made by one photographer.

**Figure 3:**
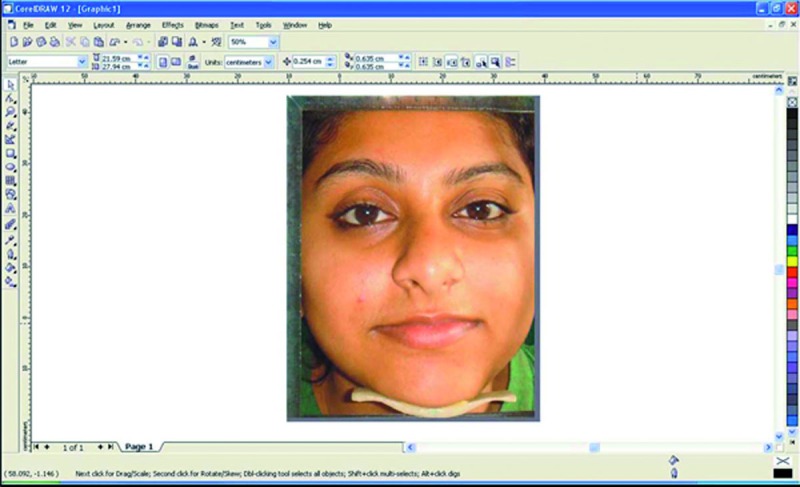
Image processing program [Corel Draw Graphics Suite 12, USA].

**Figure 4:**
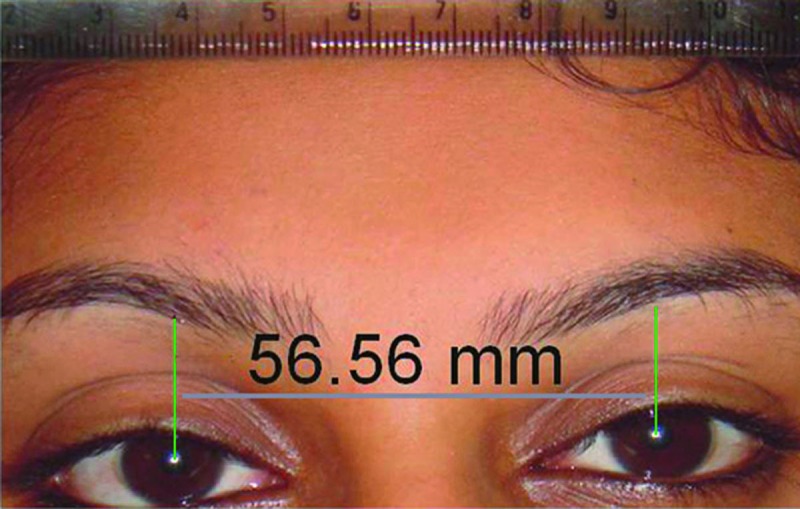
Measurement of Interpupillary distance (IPD) in mm.

This photograph was then made life-size using an image processing program [Corel Draw Graphics Suite 12, USA] (Fig. 3) and IPD from midpupil of one eye to midpupil of another was measured in millimeters (Fig. 4). All the measurements were recorded thrice by a single operator and their mean was used for further analysis to minimize the error.

For all the parameters of the study mean, standard deviation and range were calculated. Correlation was studied using Spearman’s rank correlation coefficient method. For the execution of regression command and preparation of a prediction equation to estimate VDO Statistical Package for Social Sciences [SPSS] Software Version 11.5 was used.

## Results

Descriptive statistics of the parameters studied is presented in Table 1. From  Table 1, it was observed that VDO was more in males compared to females. Also, interpupillary distance was more in males compared to females.

**Table 1:**
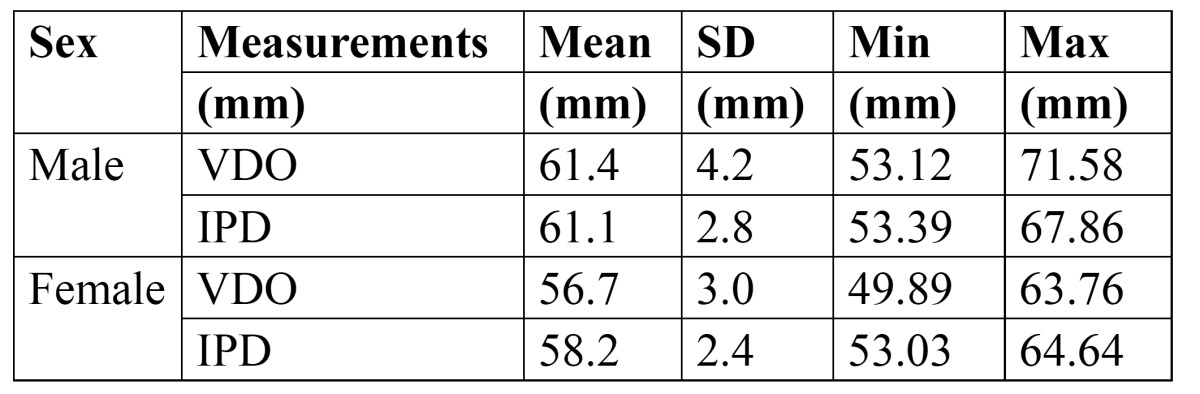
Descriptive statistics of Vertical Dimension of Occlusion (VDO) and Interpupillary Distance (IPD).

**Figure 5:**
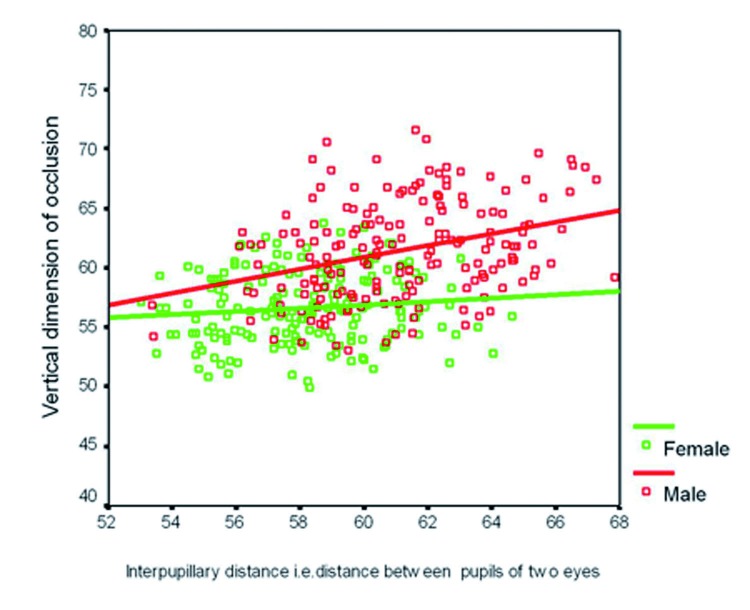
Scatter diagram along with regression lines of Interpupillary distance (IPD). versus vertical dimension of occlusion (VDO).

The coefficient of correlation [r] by Spearman’s method between the measured variable and VDO, at the probability level of 95% is presented in  Table 2. From  Table 2, it was observed that VDO is significantly and positively correlated with IPD only in males [r- 0.326] whereas in females, correlation of VDO with IPD was weak [r- 0.128]. Hence, regression analysis was performed for prediction of VDO using IPD only in males (Fig. 5).

**Table 2:**
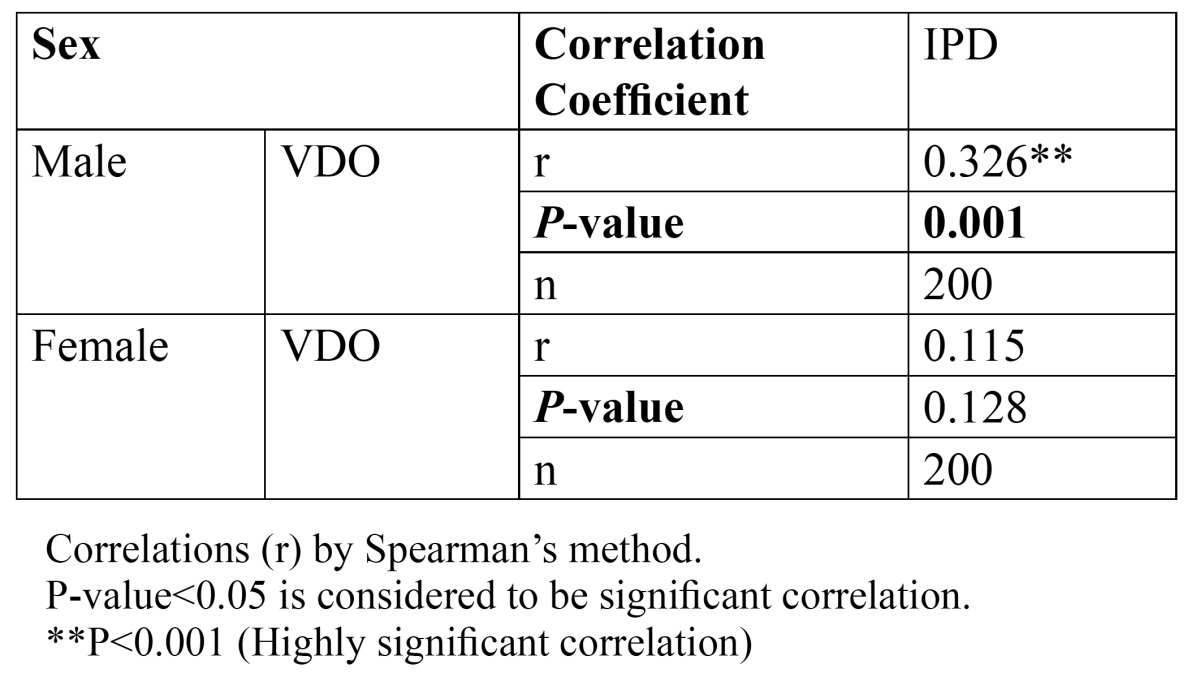
Sex specific correlations between Vertical Dimension of Occlusion (VDO) and Interpupillary Distance (IPD).

From  Table 3, it was observed that in males following regression equation was reliable to determine VDO -

**Table 3:**
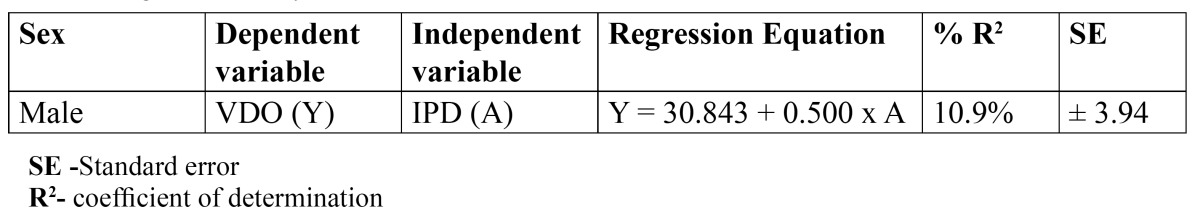
Regression Analysis in Males.

a. VDO = 30.843 + 0.500 x Interpupillary distance

Determination of VDO using regression equation for interpupillary distance had a standard error of ± 3.94 in males.

## Discussion

Loss of natural teeth and subsequent placement of an artificial prosthesis in the mouth is not a pleasurable event for any individual. Nevertheless, the agony of the patient can be lessened to some extent by providing a prosthesis which restores the original facial appearance and functions similar to natural teeth and establishing a correct VDO is one of the important steps in accomplishing this objective (2).

In the literature, many methods have been described for the estimation of VDO, but none of them is fully accepted or considered completely correct. Methods that rely on pre-extraction records like measurement of vertical and horizontal overlap of natural anterior teeth, speaking method and tattoo dot method are considered most reliable, but these records are not always available leading to difficulties in restoring occlusal vertical dimension (2). To overcome these difficulties an investigation was undertaken to find a simple yet feasible method by studying the relationship between VDO and IPD, taking into account that the growth of body parts takes place in proportion to each other.

This study revealed a sexual dimorphism with higher values for VDO as well as IPD in males compared to females. Interpupillary distance [IPD] is the facial measurement in the horizontal plane between the geometrical centers of pupillary apertures of both eyes. IPD increases till mid 20’s. The increase then slows down with negligible changes and remains fairly constant thereafter (6-10). Hence, interpupillary distance can be used as a guide in establishing VDO when patient is totally edentulous.

The interpupillary distance measured in this study showed a mean of 61.1 mm in males and 58.2 mm in females. This is in accordance with the findings of various investigators like Pointer (11) who showed a mean value of 60.14 mm in males and 57.33 mm in females. Evereklioglu *et al*. (7) found a mean IPD of 60.75 mm and 59.45 mm in males and females respectively. Swan and Stephan (12) observed a mean IPD of 63.6 mm and 59.6 mm in males and females respectively. On the other hand, values higher than the present study are reported by Gomes *et al*. (13) who found a mean value of 69.97 mm and 66.68 mm in males and females respectively. Oladipo *et al*. (14) found a mean interpupillary distance of 69.8 mm and 66.4 mm for males and females respectively. Murphy and Laskin (15) reported a mean IPD of 66.3 mm and 62.6 mm for males and females respectively.

This study revealed that interpupillary distance can be used for determination of VDO only in males with a standard error of ± 3.94. In our study, significant and positive correlation was seen between VDO and IPD only in males [r-0.326] but not in females [r-0.115]. That’s the reason why the regression equation was not derived for females. This disparity may be partly explained by the fact that IPD parameter is fairly constant in males after early middle age, but in contrast females continue to record an increase in this facial parameter into later middle age. An explanation for this hitherto unremarked feature of human facial anthropometry can be related to the gender-specific changes in underlying cranial skeletal anatomy and soft tissues of the orbital region or a combination of both (11).

The variations in the measurements found in different studies may be due to the differences in measuring techniques, ethnicities of the population and sample size studied. However, the results of this study indicated that anthropometric measurements like IPD can be helpful in estimating the VDO in males.

There are various advantages of using this method. Firstly, VDO estimation is based on objective measurements rather than subjective criteria’s such as resting jaw position (16) or swallowing (17). Moreover, estimated VDO is within the range of 2-4 mm as compared to other methods where a range of 0-14 mm is given (18,19). Other advantages include a simple, non invasive method with no radiation exposure to the patient and reproducible values for future reference.

The limitation of the study was that it was done in the subjects with class I malocclusion and other types of malocclusions were not taken into consideration. Also, the VDO measurement is difficult to record when a patient has a round facial profile with excessive soft tissue bulk under the chin. Another limitation of this method is that it did not appear useful in females. Hence, there is a scope for further research to confirm its applicability in different populations before deriving an appropriate regression equation which can be accepted universally.

## Conclusions

The results of this study indicate the possibility of using IPD to estimate VDO in male patients with variations within a range of 2-4 mm. Further studies are required to authenticate these findings and to explore the possibility in female patients.
